# Case study of radiation therapy treatment of a patient with a cardiac ventricular assist device∗

**DOI:** 10.1120/jacmp.v9i4.2851

**Published:** 2008-10-29

**Authors:** Donette E. Lasher, Jadwiga B. Wojcicka, Ronald Malcom, Lawrence L Shears

**Affiliations:** ^1^ Department of Radiation Oncology York Cancer Center York Pennsylvania U.S.A.; ^2^ Department of Radiation Oncology Carlisle Regional Cancer Center Carlisle Pennsylvania U.S.A.; ^3^ Wellspan Cardiothoracic Surgery York Pennsylvania U.S.A.

**Keywords:** ventricular assist, VAD, LVAD, EMI, external beam radiation therapy

## Abstract

A patient with a cardiac ventricular assist device (VAD) with computer‐controlled driver presented to our department for radiation therapy. The treatment plan was 4500 cGy to the rectum over 25 fractions with 15MV photon beams. All beams avoided the pump and leads. The response to electromagnetic interference (EMI) was evaluated by observing a duplicate driver in the treatment configuration as the patient's fields were delivered to a solid water equivalent phantom. Pretreatment dose assessment included calculations with Pinnacle treatment planning system, AAPM TG36 data analysis, and MOSFET measurements on the surface of the driver during the phantom irradiation. During the first patient treatment, MOSFETs were placed on the pump and leads, approximately 1cm from the left lateral treatment portal. No additional shielding was applied to the VAD. EMI was absent and the VAD operated normally during the pretreatment test and throughout the treatment course. Radiation to the driver was too low to be detected by the MOSFETS. Cumulative dose estimates to the pump were 425cGy to 0. 1cc (DVH), 368cGy (TG36), and 158.5cGy (MOSFET). MOSFET readings to the leads were 70.5cGy. External beam radiation treatment was safely delivered to a VAD dependent patient. The VAD exhibited no adverse response to EMI and doses up to 425 cGy. Our results are based on one case and further study is encouraged.

PACS number: 87.53.Dq

## I. INTRODUCTION

A 73‐year‐old male presented to our department with a T3N0M0 adenocarcinoma of the rectum requiring preoperative radiation therapy. The cancer was discovered during hospitalization for myocardial infarction and cardiogenic shock. He underwent coronary bypass grafting and was then transferred to another hospital for placement of a left ventricular assist device (VAD) (Thoratec Corporation, Pleasanton, CA). The device contains a mechanical pump that supports the left ventricle in circulating blood. It is controlled by a separate computerized driver.^(1)^ At the time of his radiotherapy simulation and treatment, the patient was dependent on the continuous operation of the VAD for cardiovascular circulation. The cardiologist's intent was to eventually wean him off the VAD.

## II. MATERIALS AND METHODS

### A. Ventricular Assist Device

**Figure 1:**
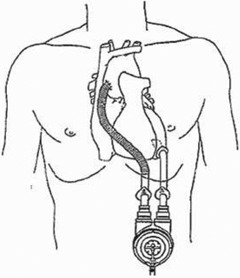
Thoratec LVAD pump as it connects to the left ventricle. The pump resides on the outside of the patient's body. (Image from Reference 1; used with permission from Thoratec Corporation.)

**Figure 2:**
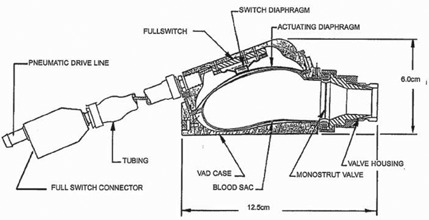
Detailed cross section of the Thoratec LVAD pump. (Image from Reference 1; used with permission from Thoratec Corporation.)

The Thoratec left ventricular assist device with TLC‐II Portable Driver is utilized for bridge to recovery or transplant after myocardial infarction and cardiogenic shock. The system consists of a pump and a pneumatic driver connected by one electrical and one pneumatic lead. The pump is surgically attached to the abdominal wall and assists the ventricle as it circulates blood. The pump resides outside the body and cannulae connect it to the ventricle as depicted in Fig. 1. It has a rigid plastic case with a small magnetic Hall Effect sensor switch. The enclosed polymer‐based blood sac is lubricated with silicone oil. (See Fig. 2) The Hall Effect sensor monitors the sac filling. As the sac fills with blood, the electric lead relays position information to the TLC‐II Portable Driver.

**Figure 3:**
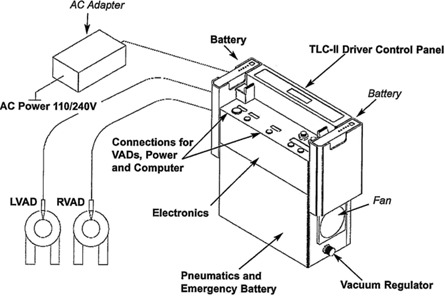
Diagram of the TLC‐II Portable LVAD Driver unit with components labeled. (Image from Reference 1; used with permission from Thoratec Corporation.)

The TLC‐II Driver (Fig. 3) contains electronic controls, a pneumatic assembly, and rechargeable batteries. The pneumatic assembly consists of an air compressor, solenoid valves for switching between filling and ejection phases, and a vacuum regulator. When the blood sac is full, the controller supplies the pneumatic lead with positive air pressure to eject the blood. During the filling cycle, it applies a slightly negative pressure. The leads between the pump and the driver are long enough to allow the patient to place the driver on a pull cart for ambulatory use, much like a small suitcase on wheels. The computer‐controlled driver is operated via an external power source and a rechargeable backup battery or two batteries alone. The electronics assembly provides all control functions for the driver, and it also contains a redundant motor drive, solenoid drive electronics, and an independent battery. In the event of a total driver and backup driver failure, the VAD can be operated with a simple hand pump.

### B. Potential harmful effects on the VAD during radiation therapy

To our knowledge, information does not exist regarding the radiation tolerance of the LVAD pump and driver, and there is no literature regarding a VAD‐dependent patient receiving radiation therapy. Pacemakers are cardiac devices more frequently encountered in radiation therapy patients. The American Association of Physicists in Medicine Task Group 34 report, “Management of radiation oncology patients with implanted cardiac pacemakers”,^(2)^ lists EMI and total accumulated dose to the device as potential concerns. The report states that although properly functioning linear accelerators do not produce problematic electromagnetic noise, patients should be monitored during the first treatment fraction. TG34 also indicates that a dose less than 200 cGy is unlikely to cause pacemaker failure.

Madigan et al reviewed the effects of EMI on ventricular assist devices in the context of defibrillation and electrocautery during surgery. They report that the possibility of EMI disrupting the operation of a VAD is highly manufacturer‐dependent.^(3)^ The extracorporeal design of the Thoratec VAD allows the timing electronics to be placed within the driver and shielded from external RF, increasing its immunity to EMI from electrocautery and defibrillation. Further, the pump itself is mechanical and should be insensitive to EMI and radiation.

### C. Functional testing of the VAD in the accelerator environment

In order to test for electromagnetic interference, a backup TLC‐II Driver unit was configured to run without the pump present. 30×18×60 cm of Plastic Water^®^ phantom was placed on the treatment couch. The backup driver was placed at the foot of the couch in the planned treatment configuration. The patient's treatment plan was executed on the phantom using the record and verify system in QA Mode. A biomedical engineer specializing in the VAD was present to monitor the driver for malfunction and alarm conditions via the intercom and video cameras. The driver's error log was reviewed after the phantom treatment.

During the phantom treatment, metal oxide semiconductor field effect transistor (MOSFET) detectors (Thomson Nielsen TN502RDM) with High Bias Setting were placed on the side of the driver closest to isocenter and under 1 cm of bolus.

### D. Patient setup and treatment plan

**Figure 4:**
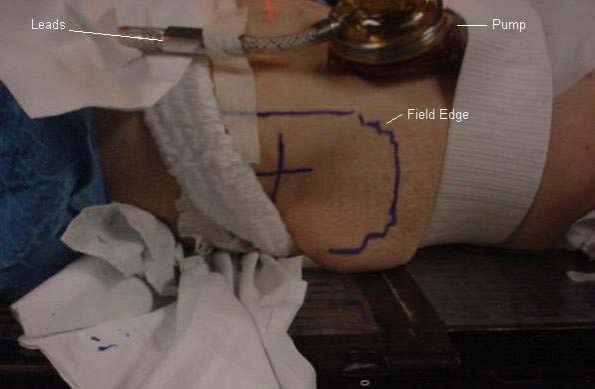
Patient setup and left lateral treatment portal.

**Figure 5:**
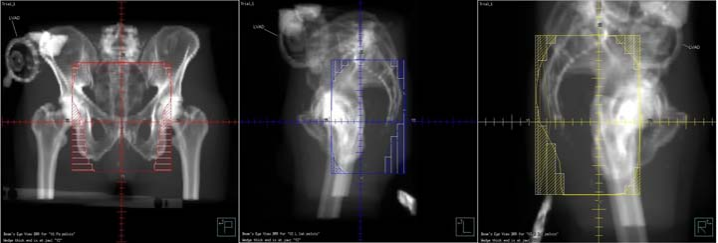
Digitally reconstructed radiographs (DRRs) of the patient's treatment fields with the LVAD pump identified. From left to right, the views are posterior to anterior, left lateral, and right lateral.

**Figure 6:**
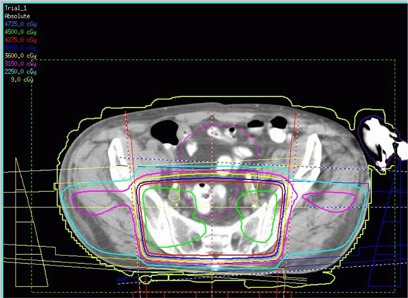
Axial view of the patient's CT‐based treatment plan. The LVAD pump is apparent on the right‐hand side of the figure. Three 15 MV photon beams are utilized, and 4500 cGy is prescribed in 25 fractions to the isocenter. The exit of the right lateral beam overlaps with the pump slightly. The isodose lines displayed are as follows: 4500 cGy (green), 4275 (red), 4050 (blue), 3600 (yellow), 3150 (pink), 2250 (teal), 9 (lime green).

The patient was positioned supine with a vacuum bag immobilizing his legs and a block strapped between his feet. The driver unit was placed on the couch at the patient's feet with a maximum lead extension (~150 cm from isocenter). The VAD pump was included in the treatment planning CT. Preoperative rectal cancer treatment planning was performed on Pinnacle v6.2b (Philips Medical Systems, Madison, WI). The prescription was 4500 cGy in 25 fractions to the isocenter with three 15 MV photon beams, a posterior to anterior and right and left laterals. A 3mm×3mm×3mm dose grid was employed, and the dose was calculated without heterogeneity corrections. The blocking on the left lateral field was designed to avoid entrance dose to the pump and the leads were taped anteriorly out of the fields (Fig. 4). The right lateral field exited through a very small part of the pump as depicted in Figs. 5 and 6. Throughout the treatment course, portal films were single exposure to the blocked field only to avoid extraneous dose to the pump and leads.

The dose to the pump was calculated by segmenting it in the Pinnacle treatment planning system and calculating the dose volume histogram (DVH). The DVH results were reviewed against dose calculations based on the treatment geometry and the out‐of‐field data in the AAPM Task Group 36 report, “Fetal Dose from Radiotherapy with Photon Beams.”^(4)^

MOSFETS were placed on the pump and leads during the patient's treatment. MOSFETS were also placed in the area closest to the left lateral treatment portal. They were covered with 1 cm of bolus material for the leads measurement. The other chips were placed between the pump and the patient's skin.

## III. RESULTS

**Figure 7:**
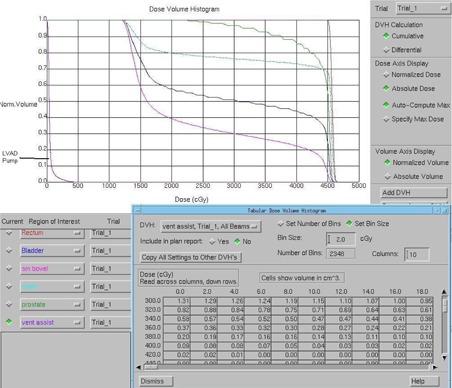
Cumulative dose volume histogram (DVH) of the pump, in both graphical and tabular form. (Generated by Philips Pinnacle version 6.2b.) The maximum dose is 425 cGy, and 1 cc of the pump receives 316 cGy.

The DVH results for the pump over the entire treatment course are displayed in Fig. 7. The maximum dose is 425 cGy, and 1 cc of the pump receives 316 cGy. Dose calculation according to the Task Group 36 out‐of‐field data is presented in Table 1. The total value of 368 cGy is consistent with the treatment plan. MOSFET measurements indicated that the leads received approximately 70.5 cGy over the treatment course (2.82 cGy per fraction). MOSFETS placed between the pump and skin registered 6.34 cGy per fraction for a total of 158.5 cGy.

**Table 1:** Dose Evaluation to the Pump via Calculation with TG‐36 Out of Field Data

	*Max Dose (cGy)*	*Equiv. Field Size (cm)*	*Distance from field edge (cm)*	*%Max dose (TG36)*	*Total (cGy)*
PA	2125	15.8	5	1.5	31.9
L lat	2548	13.3	2	2.8	71.3
R lat†	2650	13.3	−0.3	10	265.0
**Total to Pump**					*368.2*

† exit dose

MOSFETS placed on the driver during the trial run recorded negative voltage, indicating that the dose was too small to be detected. This result is consistent with the expected leakage value of ≤0.1% of the dose at isocenter, or ≤4.5 cGy over the treatment course. The driver did not alarm or malfunction during the trial run or throughout the treatment course.

## IV. CONCLUSIONS

External beam radiation treatment was safely delivered to a LVAD dependent patient. The Thoratec LVAD and TLC‐II driver did not malfunction and was not damaged during the course of radiotherapy treatment. It exhibited no adverse response to EMI. The pump safely encountered low levels (≤425 cGy) of leakage and scatter radiation, but the effect of larger doses is unknown.

The patient is dependent on the VAD for blood circulation and a device failure poses a serious health threat. Therefore, the patient's driver should be tested for EMI in a dry run of the treatment configuration before the first patient treatment. Our results are based on one case and further study is encouraged to determine device‐specific dose tolerances.
